# Study of the collaborative prevention and control mechanisms of ecological and environmental risks in China’s Yangtze River Economic Belt

**DOI:** 10.1371/journal.pone.0295017

**Published:** 2023-11-29

**Authors:** Yanhong Wang, Weiran Zhou, Lin Wang, Qianbing Ying

**Affiliations:** 1 Research Center of Agricultural Economy, School of Economics, Sichuan University of Science & Engineering, Zigong, China; 2 School of Economics, Sichuan University of Science & Engineering, Zigong, China; Soochow University, CHINA

## Abstract

The Yangtze River Economic Belt, as a globally important economic growth pole and population concentration area, has always received attention to its ecological and environmental issues. Currently, there is little research on the synergy among the ecological environment risk prevention and control mechanisms in this region. Strengthening research in this area has important scientific value for improving the effectiveness of ecological risk prevention and control and the sustainable development of the Yangtze River Economic Belt. Based on the data from 11 provinces and cities in the Yangtze River Economic Belt from 2017 to 2021, this study establishes an indicator system with benefit incentive mechanisms, risk regulatory mechanisms, and risk governance mechanisms as frameworks. By employing a composite system synergy model, this study utilizes the entropy weight method to assign weights to each indicator and calculates the orderliness and synergy of the three mechanisms separately. The results show that: (1) There are differences in the orderliness of mechanisms among the regions. The downstream area has the highest orderliness of the three mechanisms, with the middle stream area higher than the upstream area in terms of incentive mechanisms and risk governance mechanisms. (2) The orderliness of each mechanism has slight fluctuations but shows an overall upward trend, with the orderliness of regulatory mechanisms significantly higher than that of incentive mechanisms and governance mechanisms. (3) In terms of synergy, the three major mechanisms show a stable upward trend in synergy but with a relatively low degree of synergy. Based on these findings, future efforts should focus on optimizing mechanism construction and information sharing, improving incentive mechanisms, strengthening risk regulatory mechanisms, and consolidating the effectiveness of risk governance mechanisms.

## Introduction

General Secretary Xi Jinping has repeatedly stressed the overriding importance of protecting and restoring the ecological environment of the Yangtze River. He prioritizes strategies for ecological and green development and promotes environmental protection of the Yangtze River with concerted governance, focusing resources on building a green ecological corridor along the river. The Yangtze River Economic Belt is one of China’s most important economic hubs, an ecological security barrier, and an area for manifestation of ecological civilization. Protecting the ecological environment of the Yangtze River basin is, therefore, an inevitable necessity for the rejuvenation and development of China.

Yet the region is facing enormous challenges. Multiple pressures such as ecosystem degradation, environmental pollution [1], loss of biodiversity, carbon emissions, and haze pollution [2] caused by energy consumption are acting on the region. Conflicts are therefore arising between economic development and the ecological environment [3]. At the same time, global issues such as climate extremes pose a greater challenge to the ecological development of the region. In 2020, the climate in the Yangtze River Basin experienced anomalies, especially in June, when the average temperature in the basin was 1.7°C higher than the same period of the previous year. In the same year, multiple floods occurred in the Yangtze River Basin, and rainfall measured by over 200 observation stations showed historical highs in July alone. The highest temperature in the middle and lower reaches of the Yangtze River since 1961 was recorded in 2021. The director of the National Climate Center of the China Meteorological Administration, Chao Qingchen, warned that the probability of another 20-year or 100-year flood in the Yangtze River Basin would further increase. In the lower reaches of the Yangtze River, the high- temperature risk areas may exceed 50% of the total area. According to data released by the Yangtze River Water Resources Commission and the China Environmental Monitoring Station, the inflow of water to the upper Yangtze River in 2020 was nearly 30% less than that of the same period in previous years. Within the watershed, nearly one-third of the surface water is still classified as inferior to Class 5. In addition, the excessive use of agricultural fertilizers has led to a total emission of nitrogen and phosphorus that is higher than the national average. In 2020 alone, the total discharge of wastewater from the Yangtze River Economic Belt exceeded 40% of that of the country. These risks are interrelated, affecting each other in complex ways. No single action by any province or municipality can provide the best result for risk prevention and control. The cross regional and complex nature of risks in the Yangtze River Economic Belt, as well as the differences between regions, require joint effort across the entire basin for risk prevention and control. The Yangtze River Protection Act, introduced in 2020, emphasizes systemic governance [4]. To effectively control ecological and environmental risks in the Yangtze River Economic Belt, collaborative risk prevention and control methods should be constructed and further enhanced [5]. At present, China’s collaborative ecological and environmental governance suffers from unreasonable distribution of benefits and inadequate monitoring mechanisms [6]. Clarifying the ecological and environmental situation in the region, and exploring and improving the collaborative risk prevention and control methods, therefore, are an inevitable requirement for maintaining a stable ecological environment and for solving cross regional ecological and environmental problems. On this basis, the present paper constructed an index system for ecological and environmental risk prevention and control in the Yangtze River Economic Belt, and proposed corresponding optimization policies through measurement and analysis.

Researchers have conducted extensive and in-depth studies on the ecological environment of the Yangtze River Economic Belt from the perspective of collaborative development. Li et al. [7] measured green integrated factor productivity in forestry based on data from 2006 to 2021 and found that command and control of the environment can significantly contribute to green integrated factor productivity in forestry. Zhang et al. [8] pointed out that the Yangtze River basin water resource use efficiency was not adequate, with high levels of pollution and degradation of water quality and ecological function. In addition, global climate change and the relationship between water, sand, and rivers lead to new requirements for the coordinated governance of “three waters”. Their research proposed innovative methods for coordinated regulation of three waters from four aspects: in-depth analysis of the mechanisms of integration of three waters, use of models to simulate precise regulations of three water coupling, optimization of strategies for coordinated regulation of three waters, and construction of a diversified and intelligent management system for coordinated regulation of three waters. Zeng et al. [9] showed a weak positive correlation between ecological support and economic development, technological innovation, and communication services in the Yangtze River Economic Belt. They showed that the success of urban collaborative development was greater in downstream areas than in midstream areas, and greater in midstream areas than in upstream areas. They proposed reversing the ecological fragility of the Yangtze River Basin through pollution control, ecological restoration, low carbon transformation, introduction of capital, and realization of the value of ecological services. Qikai Lu et al. [10] measured carbon emissions in three urban agglomerations in the Yangtze River Economic Belt and found that carbon emissions showed a trend of increasing and then decreasing from 2008 to 2017, with emissions decreasing significantly after 2017. He [11] pointed-out that provinces and cities along the Yangtze River Economic Belt lacked management and policy regulations for collaborative governance, and constructed a diversified collaborative governance system from organization, value, market, cost, and benefits in order to integrate environmental governance and green development. Xiao et al. [12] found that there were differences in the degree of importance and responsiveness of local governments to ecological and environmental policies. Regional-level policy coordination should be promoted, and regional differences should be taken into account. Huang et al. [13] found that the synergy coefficient between the efficiency of industrial green transformation and the effectiveness of ecological civilization development in each province was low, and suggested an improvement of their synergy by increasing their importance, strengthening the ecological development in the upper and middle reaches, and improving governance. Han et al. [14] measured the coupled coordination of mineral resources, regional economy, and ecological and environmental systems in the Yangtze River Economic Belt from 2001 to 2019. The coupled coordination was greater in the downstream area than in the midstream area, and greater in the midstream area than in the upstream area. They suggested that the long-term developmental strategy should focus on individual differentiated measures and environmental protection. Xiao et al. [15] measured the degree of coordination between green innovation efficiency and ecological governance performance in 108 cities. The coordination degree increased overall, from west to east, suggesting breaking down regional administrative barriers and enhancing inter-regional synergy. Chen et al. [16] divided 30 provinces into eight regions and measured the degree of synergy for each subsystem. They found that the overall degree of synergy increased. The degree of synergy, however, was low and fluctuated greatly in the southwest economic zone (Yunnan, Guizhou, Sichuan, Chongqing, Guangxi), while it was high and increased rapidly in the eastern coastal economic zone (Zhejiang, Jiangsu, Shanghai). The middle reaches (Hubei, Hunan, Anhui, Jiangxi) were less synergistic but more stable. We propose an optimization of the synergistic environmental governance system, a collaboration on innovative technologies, and an update of governance policies. Researchers have also applied different methods to study the ecological environment of the Yangtze River Economic Belt. Li et al. [17] used a spatial lag model to empirically show that environmental regulation was conducive to high quality economic development. Leng et al. [18] showed the prevalence of PFASs midstream through water monitoring and analysis. Xu et al. [19] conducted a comprehensive evaluation of the water ecological environment of the Yangtze River Economic Belt using a weighted rank and ratio model and an adversarial explanatory structure model, and found that water ecology has not yet reached a safe and stable state. Bi et al. [20] examined the spatial and temporal changes in water supply, soil conservation, and water purification services in the Yangtze River Economic Belt from 2000 to 2018 using the InVEST (Integrated Valuation of Ecosystem Services and Tradeoffs) model and suggested that ecological conservation be tailored to local conditions. Ruchun Xiong et al. [21] used the Spatial Durbin Model (SDM) was used to analyze the spatial effects of 103 urban agglomerations from 2013–2019, and the results showed that there are spatial effects between heterogeneous environmental laws and environmental laws, and environmental regulation can significantly enhance environmental control through positive spatial effects. Yang et al. [22] developed a spatial econometric model to analyze the impact of air pollution on public health in this region and found a U-shaped relationship between air pollution and public health levels. Zhao et al. [23] analyzed the supply and demand for ecosystem services based on equivalence factors, supply and demand balance models, and quantile regression. They found a higher demand for ecosystem services in areas with developed populations and economies. Liu et al. [24] used the STIRPAT model and a dynamic panel model to examine the relationship between the urban spatial structure and the regional environment. The results indicate that a multi center urban structure can significantly reduce environmental pollution.

In general, experts and scholars have focused their research on the ecological environment of the Yangtze River Economic Belt from a synergistic perspective of the ecological environment and other subsystems. Fewer studies have focused on prevention and control of ecological and environmental risks. Even rarer are studies and discussions on mechanisms of risk prevention and control from a synergistic perspective. We performed the present study to address this lack. This study provides an important theoretical basis for the field, and may also provide new perspectives and methods for future research, further promoting the depth and breadth of research on the ecological and environmental risk prevention and control mechanisms in the Yangtze River Economic Belt. Eleven provinces or municipalities in the Yangtze River Economic Belt were selected as the research area. The synergy risk prevention and control method based on the interest incentive-risk supervision-risk governance mechanisms was constructed, the orderliness of each province or municipality as well as the synergy of the three mechanisms were measured using the composite system synergy model, and the causes were analyzed. Based on the study, we proposed an optimization of the ecological and environmental synergy risk prevention and control methods in the Yangtze River Economic Belt.

## Material and methods

### Overview of the study area

**Fig 1:**
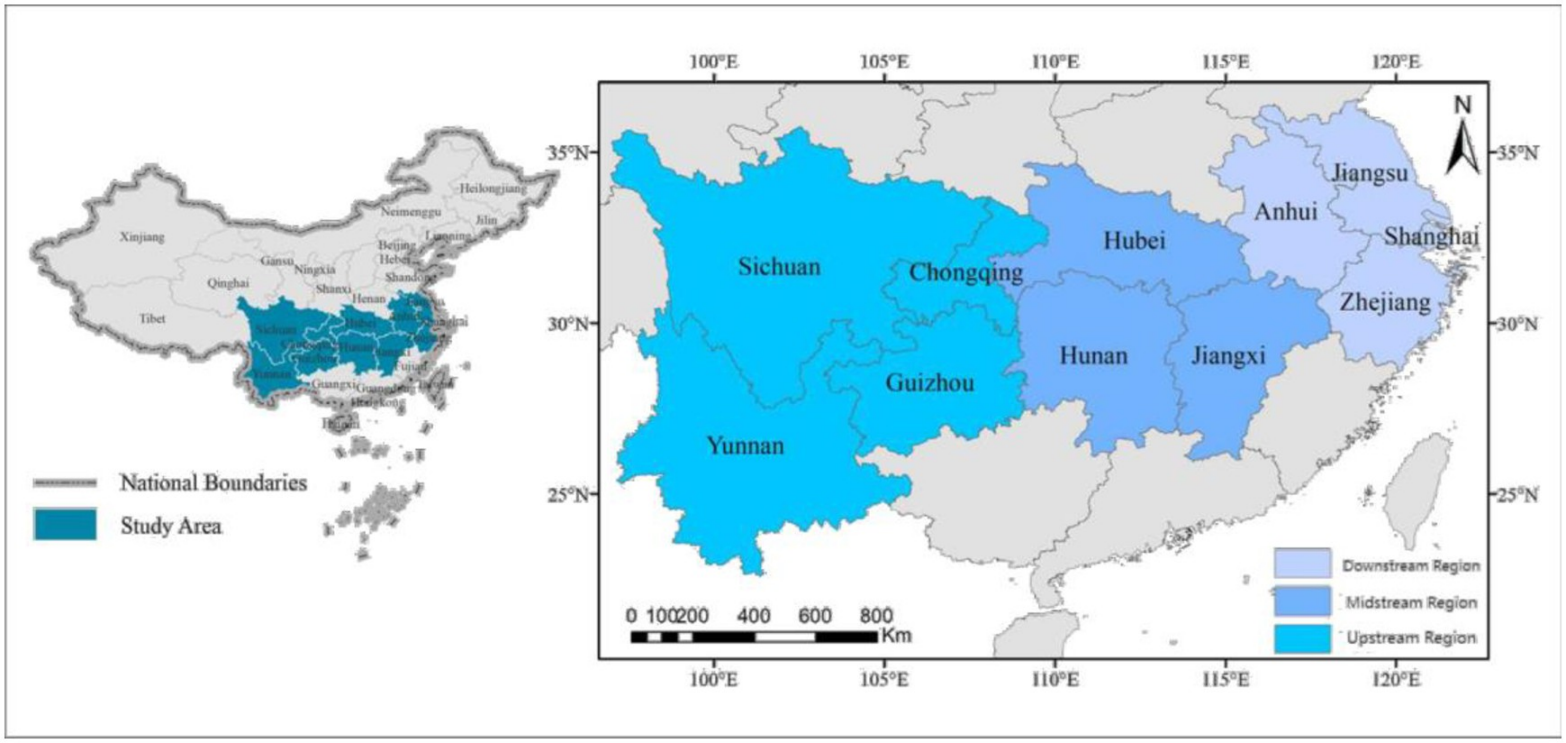
Administrative division of Yangtze River Economic Belt. This figure is modified from Yan et al [26] ’s paper: https://doi.org/10.1371/journal.pone.0260985.g002. It is not exactly similar to the original figure and is thus for illustrative purposes only.

The Yangtze River Economic Belt is an economic circle with the Yangtze River being a connecting link. It covers 11 provinces or municipalities, namely, Shanghai, Jiangsu, Zhejiang, Anhui, Jiangxi, Hubei, Hunan, Chongqing, Yunnan, Guizhou, and Sichuan (Fig 1). Yunnan, Guizhou, Sichuan, and Chongqing are found in the upstream region; Hunan, Hubei, and Jiangxi form the midstream region; and Anhui, Jiangsu, Zhejiang, and Shanghai are found downstream. The land spans an area of about 2.05 million square kilometers. It is an important strategic development zone and forms the country’s golden economic belt. The landform is complex and diverse, with forest cover rate of over 40%. The climate is tropical, subtropical, and warm temperate. As a result ecosystems are also complex and diverse. Species resources are abundant, and the biodiversity tops the seven major watersheds in China. The wetland area accounts for about 20% of the country [25].

### Model construction

In this study, the composite system synergy model was used to calculate the indices of the ecological and environmental risk prevention and control mechanisms for the Yangtze River Economic Belt. Compared with other models, the composite system synergy model is characterized by wholeness, correlation and synergy. The model considers the system as a whole and studies the complex multilayered system from a global perspective. The correlation between subsystems is understood through computational analysis, and at the same time, the synergistic relationship between the elements in the system can be assessed [27, 28]. The Yangtze River Economic Belt, as a whole, has a wide range of ecological and environmental risks, and the various elements are interrelated, so the quantitative analysis using the composite system synergy model can be better applied to this wholeness and complexity, and visualize the degree of synergism between the various elements [29].

#### Construction of indicators

Data were obtained from the 11 provinces or municipalities in the Yangtze River Economic Belt from 2017 to 2021, taken from the National Statistical Yearbook, Provincial and Municipal Statistical Yearbooks, China Energy Statistical Yearbook, China Environmental Statistical Yearbook. An index for the ecological and environmental risk prevention and control method in the region was constructed. The 11 provinces or municipalities were classified into 11 ecological and environmental risk subsystems. Each subsystem consisted of three sequential parameters, benefit incentive, risk regulation, and risk governance. Three secondary indicators were established under each sequential parameter (Table 1).

**Table 1:** Indicator system of eco-environmental cooperative risk prevention and control of Yangtze River Economic Belt.

Indicator system	Subsystem	Order parameter	Secondary indicators	Indicator nature
Collaborative Prevention and Control Mechanism for Ecological and Environmental Risks in the Yangtze River Economic Belt	Collaborative Prevention and Control Mechanism for Ecological and Environmental Risks in 11 Provinces and Cities	Benefit incentive mechanism	Investment in urban environmental infrastructure construction	positive
			Gross Regional Product	positive
			Energy conservation and environmental protection expenditure	positive
		Risk regulation mechanism	Emission of sulfur dioxide in exhaust gas	negative
			Total phosphorus emissions in wastewater	negative
			Harmless treatment rate of household waste	positive
		Risk governance mechanism	Daily treatment capacity of urban sewage	positive
			Total control rate of forest diseases, pests, and rodents	positive
			Investment completed in industrial pollution control	positive

**(1) Benefit incentive mechanism**

A sound benefit incentive mechanism not only mobilizes the enthusiasm of provinces and municipalities but also accelerates the achievement of a collaborative risk prevention and control method [30, 31]. The benefit incentive mechanism in this study mainly comprised indicators for urban environmental infrastructure investment, regional GDP, and energy conservation and environmental protection expenditure. Urban environmental infrastructure investment and energy conservation and environmental protection expenditure reflect the degree of implementation of the benefit incentive mechanism for risk prevention and control in the region. Gross regional product reflects the level of economic development and payment capacity of the region. Promoting economic development is an important reason to motivate governments to cooperate. The size of payment capacity also directly affects risk prevention and control.

**(2) Risk regulation mechanism**

Risk regulation is an extremely important part of risk prevention and control. Through risk monitoring, high risk areas can be identified, corresponding regulatory response plans can be designed according to different risk characteristics, and targeted risk management can be carried out, so as to actively mitigate risks, reduce losses, and achieve comprehensive management of ecological and environmental risks [32, 33]. In this paper, the secondary indices of the risk regulation mechanism were set as sulfur dioxide emission in exhaust gas, total phosphorus emission in wastewater, and harmless treatment of domestic waste, which reflect the implementation effect of cooperative risk prevention and control in terms of air quality, water quality, and domestic pollution, respectively.

**(3) Risk governance mechanisms**

Global environmental degradation in recent years has made countries increasingly aware of the importance of ecological and environmental risk governance. Deficiencies in governance of risk prevention and control are often an important source of risk aggregation [34, 35]. Understanding the implementation effect of ecological and environmental risk governance mechanisms in the Yangtze River Economic Belt can help improve risk governance and promote joint enforcement action in each region [36]. This indicator is, therefore, also essential in the collaborative prevention and control of ecological and environmental risks. Studying the daily urban sewage treatment capacity as a secondary indicator can help understand the water management situation. The total control rate of forest pests and rodents reflects the degree of disaster risk management. Similarly, investment in industrial pollution control can directly be reflected in risk management mechanisms.

### Calculation of composite system synergy model

**(1) rderliness of secondary indicators**

Considering the ecological and environmental collaborative risk prevention and control method of the Yangtze River Economic Belt as a complex system, let the subsystem sequential covariate secondary index be e_ij_ = (e_1j_, e_2j_, …, e_nj_), where i ∈ [1,n], j ∈ [1,11] (indicating that there are 11 provinces or municipalities in the Yangtze River Economic Belt). The orderliness of the secondary indicators is calculated as follows.

Positive indicators:

$$U\left(e_{ij}\right)=\frac{e_{ij}-\beta_{imin}}{{ɑ}_{imax}-\beta_{imin}}.$$ (1)

Negative indicators:

$$U\left(e_{ij}\right)=\frac{\beta_{imax}-e_{ij}}{{ɑ}_{imax}-\beta_{imin}}.$$ (2)

ɑ_imax_ is the maximum value of the ith secondary indicator of the jth subsystem, and β_imin_ is the minimum value. The positive indicator is also known as the effectiveness indicator, and the larger the better. The negative indicators are unfavorable indicators for the development of indicator systems. and the smaller the better.

**(2) Determining weights**

The entropy weighting method was used to assign the entropy value and entropy weight of each secondary index of the Yangtze River Economic Belt separately. The entropy weighting method is an objective assignment method, and the weights obtained are more accurate than the subjective assignment method [37]. The specific calculation is as follows:

The contribution of the ith individual to the jth indicator is first calculated:

$$P_{ij}=-\frac{X_{ij}}{\sum_{i=1}^{m}Y_{ij}}.$$ (3)

The information utility value of the jth indicator is then calculated:

$$E_{j}=-\frac{1}{lnm}\sum_{i=1}^{m}P_{ij}lnP_{ij.}$$ (4)

The entropy weights of evaluation indicators are then finalized:

$$W_{j}=-\frac{1-E_{j}}{k-\sum_{j=1}^{k}E_{j}}.$$ (5)

**(3) Subsystem orderliness**

The subsystem orderliness is calculated by integrating its corresponding ordinal parametric orderliness. The size of the subsystem orderliness is not only related to the value of the ordinal parametric orderliness but also depends on the way the ordinal parametric orderliness is integrated:

$${D(U}_{j})=\sum_{i=1}^{n}w_{i}U\left(e_{ij}\right).$$ (6)

w_i_ is the weight value, w_i_>0, $\sum_{i=1}^{n}w_{i}$ = 1. D(U_j_) denotes the subsystem orderliness. The magnitude of its value is proportional to the orderliness of the ordinal parameter, which reflects the degree of contribution to the subsystem. The larger the value, the higher the orderliness of the subsystem, and vice versa. The orderliness of the subsystem is positively related to the secondary index value.

**(4) Degree of coordination**

Assume that the order degree of the subsystem is $U_{j}^{0}(e_{j})$ at t0 and $U_{j}^{1}(e_{j})$ at t1. t0 denotes the base period, and t1 denotes the reporting period. The composite system synergy degree of the ecological and environmental risk prevention and control method for the Yangtze River economic belt is calculated as,

$$C=\gamma *\sqrt[{n}]{\prod_{j=1}^{n}{[|U}_{j}^{1}\left(e_{j}\right)-U_{j}^{0}\left(e_{j}\right)\left|\right]},$$

and

γ=1,∏[Uj1(ej)-Uj0(ej)]>0-1,∏[Uj1(ej)-Uj0(ej)]<0, (7)

where the parameter γ = 1 or -1 is used to determine the direction of coordination between the subsystems, that is, the degree of orderliness of the subsystems. When $\prod {[U}_{j}^{1}(e_{j})-U_{j}^{0}(e_{j})]<0$, the degree of synergy of each subsystem is negative, indicating that the subsystems cannot be any more synergistic with each other or cannot be synergistic at all. The value range of the composite system synergy degree is [–1,1], which reflects the degree of synergy of the mechanisms. Meanwhile, the synergy degree of the composite system is compared with that of the base period to identify changes in trends.

## Results and discussion

### Ordered degree

Because the scales of each indicator in the article construction system are different, the evaluation indicators needed to be standardized before measurement. The original data were standardized using the polar difference method, and 0.000001 was added to the standardized results to avoid any 0s. The standardized data were substituted into Eqs (1) and (2) to calculate the orderliness of each indicator in the 11 provinces or municipalities. The obtained orderliness was then substituted into Eqs (3), (4), and (5) to obtain the entropy value and entropy weight of each secondary indicator. The results of these calculations are presented in Table 2. The entropy value of each indicator was greater than 0.9, suggesting that the ecological and environmental collaborative risk prevention and control method established in this study has strong explanatory power.

**Table 2:** Indicator weights of ecological and environmental collaborative risk prevention and control method for the Yangtze River Economic Belt.

Order parameter	Secondary indicators	Information entropy e	Information utility value d	Weight (%)	
**Benefit incentive mechanism**	Investment in urban environmental infrastructure construction	0.929	0.071	12.185	34.381
	Gross Regional Product	0.924	0.076	12.993	
	Energy conservation and environmental protection expenditure	0.946	0.054	9.203	
**Risk regulation mechanism**	Emission of sulfur dioxide in exhaust gas	0.932	0.068	11.654	27.123
	Total phosphorus emissions in wastewater	0.918	0.082	14.079	
	Harmless treatment rate of household waste	0.992	0.008	1.39	
**Risk governance mechanism**	Daily treatment capacity of urban sewage	0.9	0.1	17.124	38.497
	Total control rate of forest diseases, pests, and rodents	0.972	0.028	4.803	
	Investment completed in industrial pollution control	0.903	0.097	16.57	

As can be seen from Table 2, the weights of the three major mechanisms for ecological and environmental risk prevention and control in the Yangtze River Economic Belt, in descending order, were 38.497% for the risk governance mechanism, 34.381% for the benefit incentive mechanism, and 27.123% for the risk regulation mechanism.

**Fig 2:**
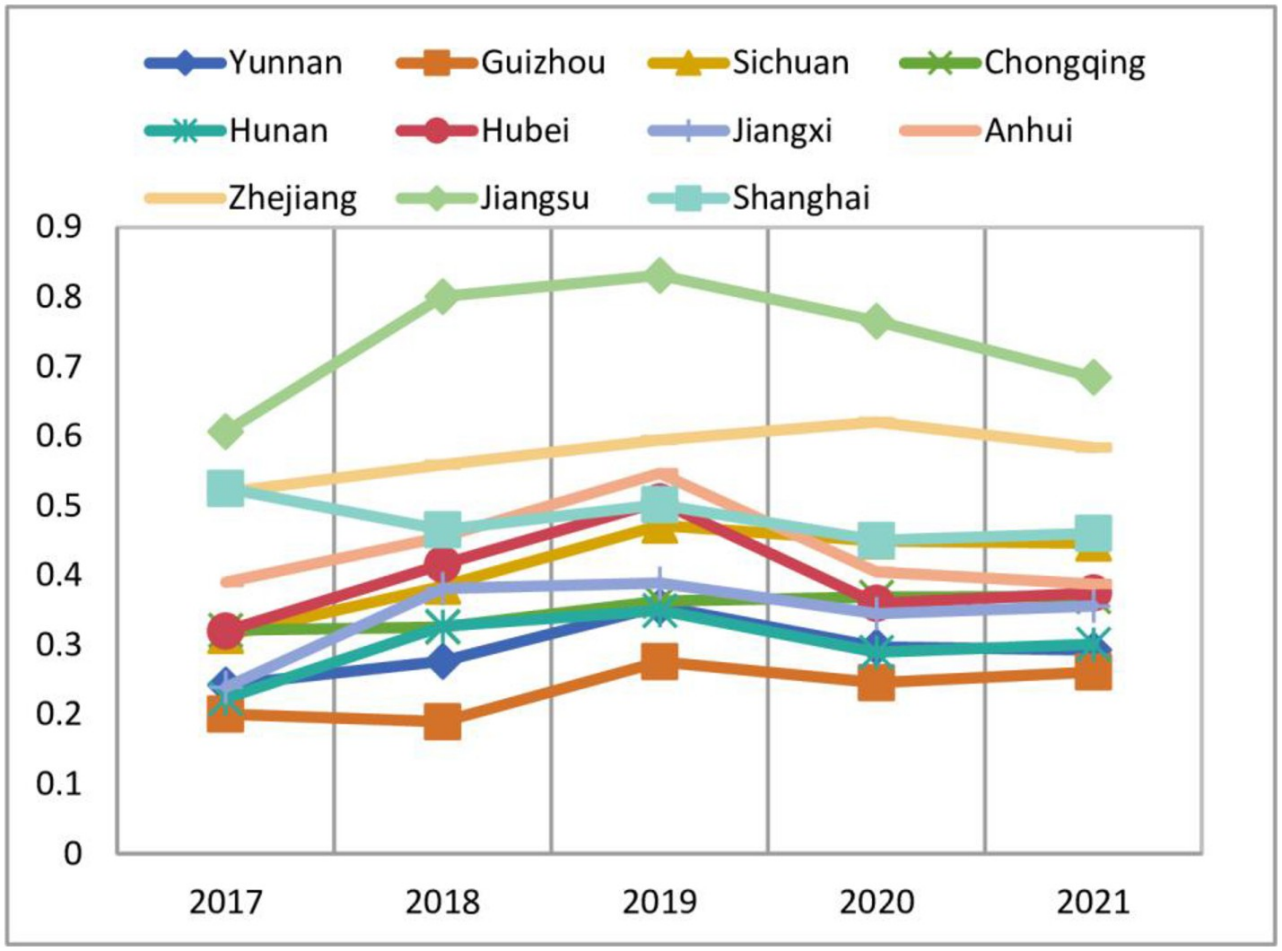
Yangtze River Economic Belt subsystem orderliness.

The orderliness of the secondary indicators combined with the weight values presented in Table 2 were substituted into Eq (6), and the linear weighting method was applied to calculate the orderliness value of each province or municipality for each calendar year. The results are shown in Fig 2.

As observed in Fig 2, Guizhou province had the lowest system orderliness, followed by Yunnan province. The provinces or municipalities with higher orderliness were Jiangsu, Zhejiang, Shanghai, and Anhui. In general, the orderliness was relatively low in the upstream area of the Yangtze River Economic Belt and higher in the downstream area. Sichuan, Chongqing, Hunan, Hubei, and Jiangxi were intermediate in terms of orderly degree. The orderliness of the 11 provinces or municipalities in the Yangtze River Economic Belt showed a fast-rising trend from 2017 to 2019. There was a slight decrease in 2020, and then the orderliness continued to rise again.

Based on the orderliness of the secondary indicators, the orderliness of the benefit incentive, risk regulation, and risk governance mechanisms in each province was calculated by applying the linear weighting method and combining with Eq (6). The orderliness of the mechanisms was divided based on the province or municipality’s location in the upper, middle, and lower reaches of the Yangtze River. The orderliness values of the three mechanisms were calculated using the geometric mean method. The results are presented in Table 3.

**Table 3:** Orderliness of three major mechanisms in the ecological upper, middle, and lower reaches of the Yangtze River Economic Belt.

Year	Upstream			Midstream			Downstream		
	Incentives	Regulatory	Governance	Incentives	Regulatory	Governance	Incentives	Regulatory	Governance
**2017**	0.0392	0.1692	0.0717	0.0952	0.1463	0.0787	0.1715	0.1812	0.1925
**2018**	0.0401	0.1725	0.0728	0.0977	0.1555	0.0824	0.1686	0.1880	0.1918
**2019**	0.0412	0.1758	0.0730	0.1035	0.1557	0.0830	0.1625	0.1909	0.1959
**2020**	0.0419	0.1758	0.0728	0.1060	0.1521	0.0853	0.1519	0.1921	0.1948
**2021**	0.0435	0.1786	0.0721	0.1077	0.1562	0.0860	0.1456	0.1991	0.1947
**Average**	0.0412	0.1744	0.0725	0.1020	0.1532	0.0831	0.1600	0.1903	0.1939

The data in Table 3 show the distribution of the orderliness of the three major mechanisms for ecological and environmental risk prevention and control in the upper, middle, and lower reaches of the Yangtze River from 2017 to 2021. From the mean value, first, the orderliness of the incentive mechanism was greater in the downstream region than in the midstream region, and greater in the midstream region than in the upstream region. The orderliness value of the downstream region was much higher than that of the midstream region, which in turn was nearly four times higher than that of the upstream region. Except for the downstream region, which had a slight fluctuation from 2020 to 2021, the orderliness of the incentive mechanism in the upstream and midstream regions showed an increment from 2017 to 2021. This suggested that the upstream and midstream regions were continuously raising their investment in ecological and environmental risk prevention and control. Second, from high to low, the orderliness of the regulatory mechanism was greater in the downstream than in the upstream region, and greater in the upstream than in the midstream region. The period 2017–2021 showed an overall increasing trend and was relatively stable, with no significant gap between regions, suggesting that provinces and municipalities in the Yangtze River Economic Belt are making continuous effort towards risk regulation, the importance of risk regulation is increasing, and the intensity and effect of risk regulation are gradually increasing [38]. Finally, the orderliness of the risk governance mechanism was greater in the downstream region than in the midstream region, and greater in the midstream region than in the upstream region. That the downstream region was much higher than both the upstream and midstream regions suggested that it had achieved better results in terms of risk governance, with the upstream and midstream regions having lower orderliness. The upstream and midstream regions would, therefore, benefit by learning more from the risk governance of the downstream region. In terms of time, the orderliness fluctuated from 2017 to 2021 except for the midstream region, which had an increasing orderliness. This suggested that there was instability in the risk governance, and the effectiveness of governance needed to be consolidated continuously.

### Synergy degree

**Fig 3:**
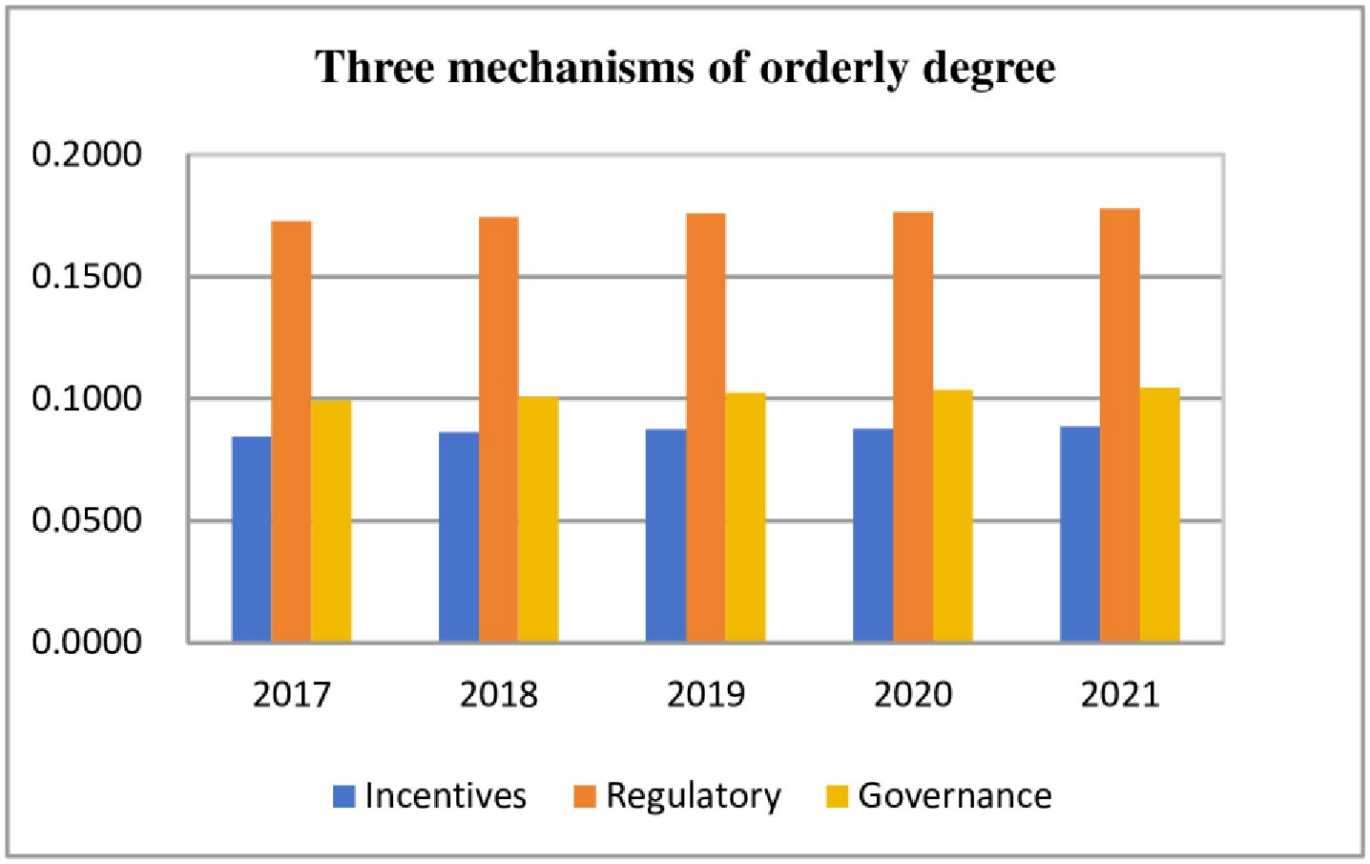
Orderly degree of ecological and environmental risk prevention and control methods of the Yangtze River Economic Belt.

**Fig 4:**
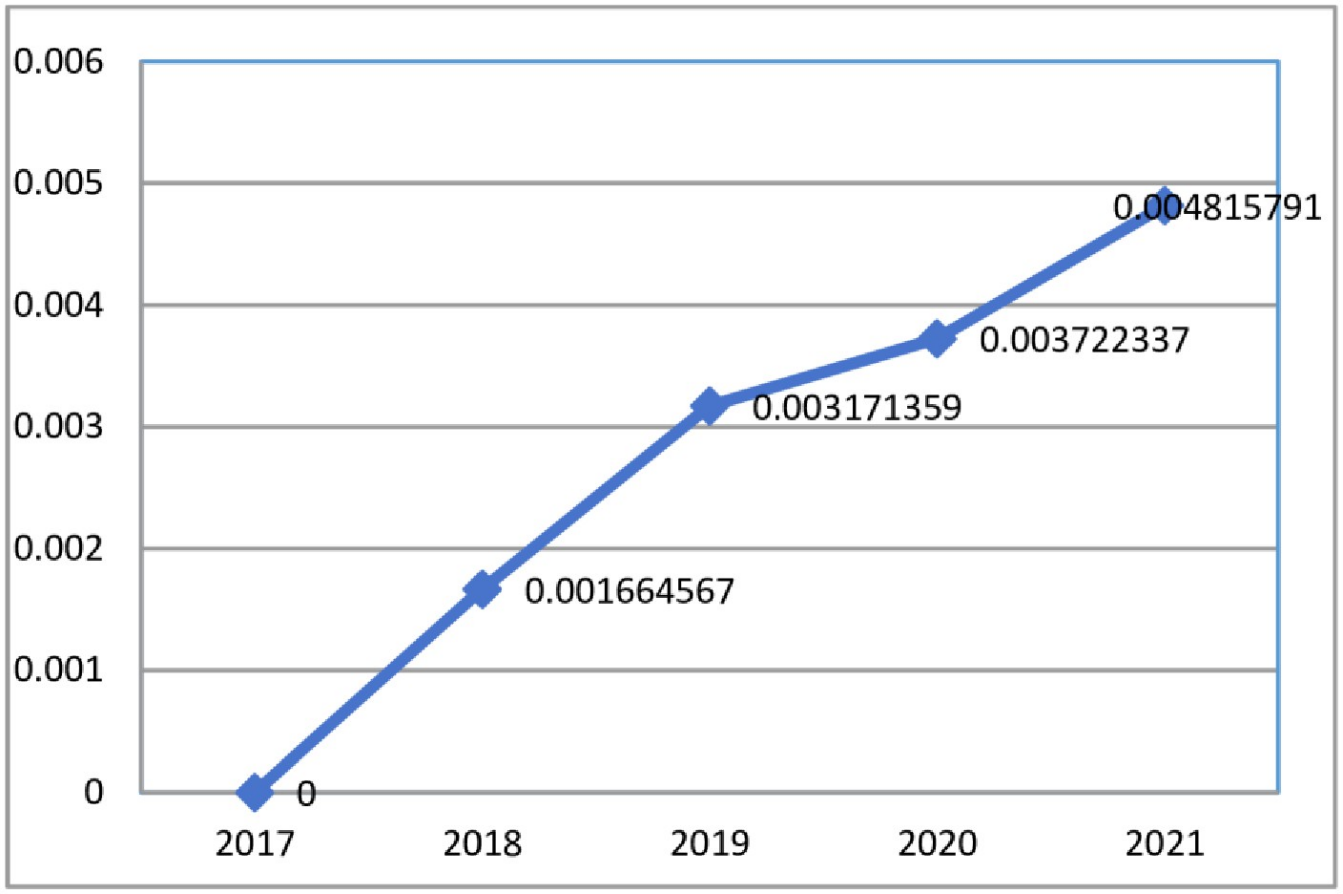
Systematic synergy of ecological and environmental risk prevention and control mechanisms in the Yangtze River Economic Belt.

First, the linear weighting method was applied to calculate the orderliness of the three major mechanisms of risk prevention and control in each province for all years, and then the geometric mean method was applied to calculate the orderliness of the three major mechanisms in the Yangtze River Economic Belt for all years. Using 2017 as the base period and applying the fixed base period method, the synergy degree of the three mechanisms in the Yangtze River Economic Belt was obtained by substituting Eq (7). The results are presented in Table 4, Figs 3 and 4.

**Table 4:** Orderliness and synergy of ecological and environmental risk prevention and control methods in the Yangtze River Economic Belt.

Year	Incentives	Regulatory	Governance	Synergy
**2017**	0.0843	0.1728	0.0991	-
**2018**	0.0862	0.1745	0.1007	0.0017
**2019**	0.0874	0.1759	0.1025	0.0032
**2020**	0.0876	0.1765	0.1036	0.0037
**2021**	0.0885	0.1778	0.1046	0.0048

According to Fig 3, the orderliness of the three major mechanisms in the Yangtze River Economic Belt, in descending order, was as follows: the risk regulation mechanism was greater than the risk governance mechanism, and the risk governance mechanism was greater than the benefit incentive mechanism. Additionally, the orderliness of the risk regulation mechanism was significantly higher than that of the other two mechanisms, being about two times higher than that of the benefit incentive mechanism. The orderliness of the three mechanisms showed a gradual increase from 2017 to 2021, but the growth trend was slow, and the overall orderliness value was low, suggesting that there is still much room for optimization in the implementation of the three mechanisms for ecological and environmental risk prevention and control in the Yangtze River Economic Belt and that the construction needs to be continuously strengthened.

As shown in Fig 4, the synergistic effects of the three mechanisms showed an overall stable upward trend from 2017 to 2021. This suggested that since General Secretary Xi Jinping introduced policies related to promoting the development of the region in 2016 and 2017, the provinces and municipalities have taken action and achieved results in terms of regional synergy risk prevention and control. The rising trend from 2019 to 2020 was relatively flat compared to the other years. The low value suggested that the three major mechanisms for ecological and environmental risk prevention and control in the Yangtze River Economic Belt had weak synergy and that linkage and synergy of the individual mechanisms needed to further be strengthened.

## Conclusions

### Research conclusion

This study calculates and analyzes the synergy degree of the ecological and environmental risk synergistic prevention and control mechanism of the Yangtze River Economic Belt based on the relevant indicator data of 11 provinces and cities in the Yangtze River Economic Belt from 2017 to 2021, and the findings further support the findings of previous researchers in this field [39–43]. First, the indicator system related to ecological environment shows a general increasing trend of synergy degree in the synergy measure, but the synergy degree is generally low. Secondly, ecological environment risk prevention and control is geographically characterized by strength in the east and weakness in the west. This study provides new evidence on the basis of previous studies, and by analyzing and calculating the breakdown to form the data of each province and the upper, middle and lower reaches, it can more intuitively understand the ecological risk prevention and control situation in each region, thus providing a more accurate reference for policy making. Specifically, the study draws the following conclusions:

#### Low degree of synergy among the three mechanisms

The degree of synergy between the benefit incentive, risk regulation, and risk governance mechanisms showed a slowly increasing trend, but the overall degree of synergy was low, and the synergy of risk prevention and control among provinces and municipalities was weak. The reasons for this were, first, at the policy level, the provinces and municipalities were not specific enough in the formulation of policies for ecological and environmental risk prevention and control, resulting in inconsistent implementation and poor system execution. Some mechanisms were more macro-level and simple, some were for the Yangtze River Economic Belt in general, and some were for local watersheds or single elements such as water or air. The lack of collaborative and unified principles and objectives among provinces and municipalities, and the incompatibility between mechanisms, resulted in docking difficulties and ineffective synergy [44, 45]. Second, at the information level, there were a lack of professional information platforms for risk prevention and control, which limited the rapid transmission or sharing of information between provinces and municipalities. In addition, most of the existing data were relatively old, less timely, and less comprehensive, challenging the timely communication between provinces and municipalities to prevent and handle risk events. The scope of information sharing processes was also unclear, with methods for sharing risk prevention and control technologies and experiences being limited. This, in turn, affected the overall synergy risk prevention and control of the Yangtze River Economic Belt to a certain extent.

#### Regional imbalance of benefit incentive mechanism

Benefit incentives were significantly higher in downstream areas than in midstream and upstream areas. The downstream region had the highest benefit incentive mainly because it had rich resources, had the most developed economy, and had a higher level of green development [46]. The ecological environmental protection results could be directly transformed into economic benefits, the demand for ecological environmental protection was more urgent, and there was sufficient financial investment in ecological environmental risk prevention and control. The benefit incentive was, therefore, more obvious in the downstream areas. In contrast, the midstream and upstream areas were relatively backward in terms of economic development. This was especially the case for the upstream areas. The geomorphology and geographical location of this region limited its economic development [47], and coupled with government policy in favor of economic development, there was less investment in and motivation for ecological environmental protection. Implementation of the benefit incentive mechanism was, therefore, limited. Urban agglomerations in the midstream region need to balance economic development with ecological and environmental protection. The ecological pressure and resource constraints are relatively large [48]. Although the demand for benefit incentives was higher, however, its implementation was limited by resources and funds. In conclusion, the imbalance of economic development and ecological and environmental pressure, and differences in resource endowments, have led to differential input in terms of risk prevention and control across the basins.

#### Need for continuous strengthening of the risk regulation mechanism

Development of the risk regulation mechanism in each basin was relatively smooth, but the overall degree of regulation was low and still needs continuous attention. Our analysis showed that the risk regulation mechanism received attention, and the intensity of regulation was increasing, but the degree of regulation was still low. The level of economic development and technology in each region was not synchronized, and the ability to monitor environmental risks varies, making it difficult to synchronize regulation. Downstream areas were more economically and technologically developed, and the corresponding regulatory mechanism was more effective. In upstream areas, lately, due to resource endowment, and the ecological and environmental complexities and risks, risk regulation and investment have been gaining increasing attention year by year. This has achieved certain results. The economy of the midstream region, for historical reasons, was mainly driven by industrial development, with a large number of chemical enterprises present along the rivers, high wastewater emissions, and serious air pollution, making it difficult to supervise prominent risks [49–51]. Environmental risk regulation was a long-term dynamic process and is lagging behind. The lack of governance in the upstream region also affected the regulatory governance in the midstream region. As a result, implementation of risk regulation mechanisms in the midstream region was less effective.

#### Need for consolidation of the risk management mechanism

The ecological and environmental risk management of the Yangtze River Economic Belt still varied and fluctuated among basins. The overall collaborative method for risk management was not effective enough and lacked long-term vision. There was still a lack of proactive and targeted risk resolution methods and intervention mechanisms [52]. First, the central goal of risk management was not clear, leading to reduced effectiveness in governance. Second, because ecological and environmental risk management was a long-term process, and the effectiveness may fluctuate in the short term, long-term tracking and adjustments were required. Downstream areas were more technologically advanced due to geographical factors, and joint enforcement and governance actions were carried out more frequently. Risk management was, therefore, the most effective in this region. The fluctuations in the downstream areas may be because air quality cannot be guaranteed due to the high urbanization, tailpipe emissions, and urban population density. The effect of the governance mechanism in the midstream region was gradually increasing, probably because the midstream region was dominated by industrial water pollution. In recent years, the government has made great effort targeting risks from industrial enterprises along the river, carrying out special rectification actions for sewage treatment facilities, controlling the rise of polluting enterprises, and eliminating backward enterprises. Water pollution remediation has, therefore, made great progress. The state-controlled cross-sectional water quality (Grade 1 to 3) of the Yangtze River went from 82.8% in 2017 to 97.1% in 2021. The risk management techniques in the upstream areas were relatively backward. The insufficient linkage between provinces and municipalities coupled with frequent natural disasters meant that the risk management mechanism was less effective and fluctuating.

### Optimizing policies

The Yangtze River Economic Belt is a pioneering demonstration for the construction of an ecological civilization in China. Our study proposes the following recommendations for the ecological and environmental problems faced by the Yangtze River Economic Belt, including prominent risks and the lack of effective collaborative methods for risk prevention and control.

#### Optimize mechanism building and information sharing

First, the mechanism construction should be optimized. A collaborative mechanism for ecological and environmental risk prevention and control should be established in each region of the Yangtze River Economic Belt. It is not sufficient to have a macroscopic system for ecological risk prevention and control. A corresponding and feasible system based on the specific conditions of each province and municipality should be developed, taking into account each of their situations and then integrating with macroscopic policies [53, 54]. The principles and objectives of ecological and environmental risk prevention and control should be clarified, and collaboration between the cross-provincial and municipal methods should be deepened. The implementation of the policy system should be strengthened enabling policies to be put into practice. Second, information sharing should be optimized. A unified ecological and environmental risk information platform should be established for the Yangtze River Economic Belt to achieve rapid information circulation and sharing. Artificial intelligence and big data should be made use of to achieve real-time sharing of information related to ecology and environment to better handle risk events [55]. Cooperation and communication between provincial and municipal governments and departments should be strengthened; systems and norms related to information sharing should be developed; and the scope, process, and responsibilities of information sharing should be clarified [56–58]. Experience and technology sharing among provinces and municipalities should be improved to improve overall synergy in ecological and environmental risk prevention and control.

#### Improve the benefit incentive mechanism

First, according to the economic, social, and resource conditions of different regions, differentiated incentive policies should be developed to suit the actual requirements of each region [59, 60]. For the upstream region, focus should be on economic development while increasing investment in environmental protection governance and encouraging private capital enterprises to participate in environmental protection governance. The midstream regions should balance the relationship between economic development and environmental protection governance. Enterprises should be encouraged to invest in research, development, and renovation of environmental technology; environmental protection costs should be reduced; utilization rate of resources should be improved [61]; industrial transformation should be promoted; and manufacturing industries should be encouraged to shift to the high-end [62]. Downstream areas should continue to invest in environmental protection governance, but further breakthroughs can be made for risk prevention and control technologies. Second, an ecological compensation system should be implemented. Pilot ecological compensation should be promoted in areas with risks, appropriate rewards should be provided to areas with significant ecological environmental protection methods, special financial subsidies should be increased to support the relatively economically backward upstream and midstream areas, environmental tax policies should be optimized, and the focus of funding arrangements should be clarified [63].

#### Strengthen the risk supervision mechanism

First, the ecological and environmental risk regulation policy system should be improved to ensure that the regulatory policy is targeted and its implementation effective, while promoting regulatory informatization, improving regulatory efficiency, strengthening cross-regional regulatory cooperation and resource sharing, and realizing joint law enforcement and collaborative supervision. Second, a perfect monitoring system should be established. The regulation of pollutants in the Yangtze River Economic Belt should be strengthened [64], focusing on agricultural-surface source pollution for upstream areas, for example, the specific amount of fertilizer and agricultural film used and the treatment of poultry manure, as well as on the development of resource-intensive industrial clusters centered on Sichuan and Chongqing [65]. Cities in the midstream region should continuously monitor water quality and the discharge of sewage, and prevent the risk of water pollution. The downstream areas should mainly strengthen regulations around air pollution, continuously monitoring haze weather and inhalable particulate matter. At the same time, regular quality reports should be issued for water quality and air quality monitoring. The investment in monitoring facilities should, therefore, be increased to promote the technological development of monitoring equipment and to improve the accuracy and timeliness of monitoring data.

#### Consolidate the effectiveness of the risk management method

First, the roles and responsibilities of participants in the ecological and environmental risk governance should be clarified, communication and information sharing should be strengthened, the synergy of all parties should be formed, and the effectiveness of ecological and environmental risk governance should be incorporated into the government’s annual target assessment index system. Second, continuous investment in risk management funds should be made to ensure a stable advancement of the management projects, and social entities should be encouraged to actively participate in environmental management and share a certain amount of management funds. At the same time, the research and innovation of ecological and environmental risk management technology should be strengthened to improve the effectiveness of governance. Additionally, the tracking and evaluation of risk management should be strengthened, the strategy should be adjusted in time to ensure its sustainability, and the focus of management should be adapted to specific regions [66]. Upstream areas should improve disaster warning and emergency response capabilities, and increase investment in governance equipment and technology. The midstream region should consolidate the effectiveness of water governance, strengthen remediation efforts as well as development and use of water-saving technology, and improve the efficiency of water resource utilization [67], thereby promoting industrial transformation and upgrading [68–70]. Downstream areas should promote public transportation and encourage the research, development, and use of new energy sources [71], thereby continuing to play an active role in carbon reduction in urban parks [72].

### Research limitations and future research

This paper measured the ecological and environmental collaborative risk prevention and control method of the Yangtze River Economic Belt and proposed optimized countermeasures. There were, however, some shortcomings. In the construction of the index system for the ecological and environmental collaborative risk prevention and control method of Yangtze River Economic Belt, we mainly focused on the interest incentive, risk supervision, and risk governance mechanisms. The number of secondary indicators was small, and the indicators were not comprehensive enough. We did not pay sufficient attention to other mechanisms of ecological and environmental risk coordination and risk prevention and control for the Yangtze River Economic Belt, such as the risk emergency and risk assessment mechanisms. Future research can improve the comprehensiveness of the proposed index system.

## Supporting information

S1 Data (XLSX)
